# Refinement and Enhancement of *Agrobacterium*-Mediated Transient Transformation for Functional Gene Examination in Mulberry (*Morus* L.)

**DOI:** 10.3390/genes15101277

**Published:** 2024-09-28

**Authors:** Rongli Mo, Na Zhang, Changyu Qiu, Sheng Huang, Wei Wei, Chaohua Zhang, Dan Liu, Qiang Lin

**Affiliations:** 1 Guangxi Zhuang Autonomous Region Sericultural Technology Promotion Station, Nanning 530007, China; moqianchun1987@163.com (R.M.); zhangnayuanwu@126.com (N.Z.); changyuqiu2008@163.com (C.Q.); gxcyhs@163.com (S.H.); gxcanyeweiwei@126.com (W.W.); zhangchaohua2023@163.com (C.Z.); liudanpl@163.com (D.L.); 2 Guangxi Research Academy of Sericultural Science, Nanning 530007, China; 3 Guangxi Key Laboratory of Sericultural Genetic Improvement and Efficient Breeding, Nanning 530007, China

**Keywords:** mulberry, *A. tumefaciens*, transient transformation, vacuum infiltration

## Abstract

**Background:** Mulberry (*Morus* L.), a vital perennial woody plant with significant economic importance, is utilized for silkworm rearing, human consumption and medicinal use. The availability of mulberry’s whole-genome sequencing data has underscored the demand for an effective, user-friendly, and high-throughput protocol to facilitate the elucidation of gene functions. **Methods and Results:** In this investigation, we established a transient transformation approach using *Agrobacterium tumefaciens*-mediated sonication followed by vacuum infiltration in mulberry tissue culture seedlings. Simultaneously, we optimized the transformation conditions, including mulberry genotypes, *A. tumefaciens* strain, acetosyringone concentration, bacterial density, sonication time, and days after agroinfiltration. These optimizations aimed to achieve heightened transformation efficiency, employing GFP as a reporter gene to monitor transformation events. The optimized method included the use of an infiltration medium (10 mM MgCl_2_, 10 mM MES (2-(N-morpholino)ethanesulfonic acid sodium salt), 150 μM acetosyringone, and OD600 0.5 of *A. tumefaciens* LBA4404) supplemented with the surfactant 0.02% Silwet L-77, with 20 s sonication followed by 20 min vacuum infiltration (0.07 MPa). Among the four mulberry genotypes, ‘Taiguo’ was the most responsive genotype and produced the highest levels of *GFP* expression at 7 d after infiltration. Furthermore, the optimized transient transformation approach has been proven to be successfully applicable for transiently overexpressing *MaANS* and *MaDFR* in mulberry fruits of ‘Taiguo’, in vitro, which distinctly enhanced fruit coloring and significantly increased anthocyanin accumulation, respectively. **Conclusions:** In summary, we devised a dependable, stable and highly efficient transient transformation approach suitable for rapid gene function examination in mulberry leaves and fruits, in vitro.

## 1. Introduction

Mulberry (*Morus* L.), boasting significant economic importance and deeply rooted in Chinese history, primarily serves as the key source of leaves for silkworm (*Bombyx mori* L.) rearing, contributing to the production of exquisite silk fibers [1,2,3]. The mulberry tree exhibits a robust and intricately networked root system, imparting resistance to drought, flood, and sandstorms, and facilitating water and soil conservation [4,5,6]. Renowned for its rich content of proteins, flavonoids, carotene, amino acids, pectin, carbohydrates, fiber, minerals, and vitamins, the mulberry fruit holds the status of a third-generation fruit, enjoying a lengthy history of use in traditional Chinese medicine as well as being an edible fruit [2,7,8,9]. Recent clinical application studies have unveiled different pharmacological effects, including blood sugar level reduction, blood lipid level decrease, and anti-aging properties [10,11,12,13,14,15]. With the successive release of genomes for *Morus notabilis* [16,17], *Morus alba* [18], *Morus indica* [19], and *Morus atropurpurea* [20] since 2013, mulberry research has seamlessly transitioned into the post-genomic era, setting the stage for in-depth molecular mechanism examination and utilization of mulberry’s outstanding traits, and the comprehensive development and deployment of mulberry resources. Consequently, the establishment of an efficient and stable genetic transformation system for mulberry trees has been taken as a necessary strategy to verify the gene functions in the post-genomic era, bearing immense significance for elevating the quality and efficiency of the mulberry trade and advancing select mulberry disciplines.

In the realm of plant studies, stable genetic transformation and transient transformation mediated by *A. tumefaciens* have become widely adopted for gene function verification in various crops, including citrus [21], apple [22,23], poplar [24,25], and more. The initial report on leaf disc transformation of mulberry (*M. alba* L.) through *Agrobacterium*-mediated methods dates back to Machii [26]. Subsequently, *A. tumefaciens*-mediated stable genetic transformation of *M. indica* cv. K2 has been constructed and improved [27,28,29]. Despite numerous reports on the efficient transformation of various explants in mulberry [30,31], achieving stable and efficient genetic transformation across mulberry species is still a challenge. Limitations stem from the high antibiotic content/selection pressure, leading to callus browning, genotype-dependent adventitious bud regeneration, and a transformation system confined to specific mulberry genotypes, such as *M. indica* cv. K2 [27,28,32] and *M. indica* cv. M5 [29]. Additionally, the existing transformation system faces constraints related to low efficiency and high labor costs, rendering it unsuitable for rapid and high-throughput gene function examination.

Conversely, in comparison to stable genetic transformation, the transient transformation mediated by *A. tumefaciens* has gained widespread popularity in characterizing gene functions in woody plants, offering numerous advantages, including simplicity, a short timeframe, high efficiency, easy implementation, and cost and labor savings [33,34,35,36,37,38,39]. To date, only one study has documented the development of a transient transformation system in mulberry seedlings using *A. tumefaciens* via syringe [40]. Furthermore, plant virus-mediated transient transformation approaches, like VIGS, for gene functional confirmation in mulberry have been explored [41]. However, syringe infiltration limitations related to leaf architecture hinder *Agrobacterium* cell access to plant tissues, leading to unstable transformation efficiency [34,35]. Prior research advocates the use of vacuum infiltration for *Agrobacterium*-mediated transient transformation in plants, overcoming leaf architecture challenges and enhancing foreign gene expression compared to syringe infiltration [33,42,43,44,45]. Additionally, sonication has been employed to boost *Agrobacterium* delivery, creating numerous microwounds in target tissues through cavitation, facilitating more efficient bacterial access to internal structures [45,46,47,48].

This study presents the development of a straightforward, highly efficient, and user-friendly *Agrobacterium*-mediated transient transformation approach in mulberry tissue culture seedlings, employing *GFP* as a reporter gene and utilizing sonication and vacuum infiltration. Furthermore, key factors influencing the efficiency of transient transformation, including *Agrobacterium* strains, mulberry genotypes, sonication treatment time, acetosyringone concentration, bacterial density (OD_600_), and days after agroinfiltration, were thoroughly assessed and enhanced. To examine the system’s efficiency, *MaANS* (anthocyanidin synthase) and *MaDFR* (dihydroflavonol reductase), involved in anthocyanin accumulation, were transiently overexpressed in mulberry fruit, causing a substantial enhancement of fruit coloring and anthocyanin levels.

## 2. Materials and Methods

### 2.1. Plant Material

The investigation utilized tissue culture shoots derived from four distinct mulberry genotypes: ‘Taiguo’ (*M. alba* L. 2n = 2X = 28), ‘Aoyu’ (*Morus multicaulis* Perr. 2n = 2X = 28), ‘8632’ (*M. multicaulis* Perr. 2n = 2X = 28), and ‘Yaosang’ (*Morus nigra* L. 2n = 22X = 308). The in vitro cultures were nurtured under controlled conditions at 24–26 °C with continuous 16 h illumination provided by white fluorescent tubes (2000–3000 lux). Subculturing was performed every 5 to 6 weeks. The subculture medium composition varied based on the genotype: the tissue culture shoots of ‘Taiguo’, ‘Aoyu’, and ‘8632’ were maintained on Murashige and Skoog’s (MS) medium (pH 5.8, composed of 6.5 g·L^−1^ agar, 1.0 mg·L^−1^ 6-benzyladenine (6-BA), 30 g·L^−1^ sucrose, and 0.5 mg·L^−1^ naphthaleneacetic acid (NAA) [49,50]. In the case of ‘Yaosang’, the Driver and Kuniyuki Walnut (DKW) medium was employed, enriched with 30 g·L^−1^ sucrose, 3.0 mg·L^−1^ zeatin (ZT), 3.0 mg·L^−1^ 6-BA, 0.5 mg·L^−1^ NAA, and 6.5 g·L^−1^ agar with the final pH set at 5.8. Here, leaves from actively growing tissue culture shoots, sampled after 4 weeks in subculture, served as the primary material for all experimental procedures.

The mulberry fruits of ‘Taiguo’ were used in this study. The mulberry trees were grafted onto ‘Guisangyou 12’ (*M. atropurpurea* Roxb.) rootstocks using pocket grafting in 2018 and were planted at a spacing of 1.5 × 3.5 m in the Mulberry Repository of Hubei Province (latitude: 30°48′7″, longitude: 114°33′4″, and altitude: 28 m) in Wuhan, China. The fruits were harvested 10 d after flowering (DAF) for transient genetic transformation experiments.

### 2.2. Formulation of Agrobacterium Strains Used in Infiltration

The pMV2-*GFP* vector [34], a construct featuring the *GFP* reporter gene regulated by the CaMV 35S promoter, was introduced into three distinct strains of *A. tumefaciens*, namely LBA4404, EHA105, and GV3101. In accordance with established protocols [34,35], an actively growing *A. tumefaciens* cell culture underwent a 1 mL transfer into 50 mL of LB media for an overnight incubation at 28 °C. Subsequently, the *Agrobacterium* cells were harvested via centrifugation (4000× *g*, 10 min) and eventually resuspended to an OD_600_ of 0.75 using the infiltration medium (composed of 10 mM MES, 10 mM MgCl_2_, 0.02% Silwet L-77, and 150 μM acetosyringone) [45]. It is noteworthy that the *Agrobacterium* cell suspension underwent a 3 h incubation at room temperature before the infiltration process.

To assess the impact of different *Agrobacterium* strains and mulberry genotypes on transient transformation efficiency, a vacuum (0.07 MPa) was applied for 20 min intervals, consistent with established procedures [45]. The suspensions of *A. tumefaciens* strains GV3101, EHA105, and LBA4404, each carrying the pMV2-*GFP* vector, were injected into the leaves of the ‘Taiguo’ genotype (Table S1). Additionally, the *A. tumefaciens* strain LBA4404 suspensions were introduced into the leaves of four mulberry genotypes: ‘Taiguo’, ‘Yaosang’, ‘Aoyu’, and ‘8632’, respectively (Table S1). Subsequently, the infiltrated leaves underwent cocultivation with *Agrobacterium* on MS medium and DKW medium (‘Yaosang’), as described above, for a duration of 4 d at 28 °C with a continuous 16 h illumination provided by white fluorescent tubes (2000–3000 lux). Following this incubation period, one part of the infiltrated leaves from ‘Taiguo’, ‘Yaosang’, ‘Aoyu’, and ‘8632’ were subjected to GFP imaging, and the other part was used to assess GFP fluorescent protein determination.

One part of the infiltrated leaves from ‘Taiguo’, ‘Yaosang’, ‘Aoyu’, and ‘8632’ was used for GFP fluorescence detection, and the other part was used for GFP protein determination.

### 2.3. Optimization of Experimental Parameters

To comprehensively assess the myriad factors influencing transient transformation efficiency, an extensive exploration of parameters was conducted in mulberry leaves of the ‘Taiguo’ genotype using *A. tumefaciens* strain LBA4404-mediated vacuum infiltration. In total, each treatment involved the analysis of ten single-leaf replicates. The investigated parameters encompassed bacterial density (OD_600_ 0.5, 0.75, and 1.0) in the infiltration medium, comprising 150 μM acetosyringone, 10 mM MES, and 10 mM MgCl_2_, as well as acetosyringone concentration (50, 100, 150, and 200 μM) in the infiltration medium, comprising 10 mM MES, 10 mM MgCl_2_, and a final OD_600_ of 0.75 (Table S1). Additional parameters included sonication time (0, 10, 20, 30 s, respectively) and days after infection (4, 7, 10, 15 d, respectively) (Table S1). With the exception of the ultrasonic treatment experiment, all leaves were initially subjected to a 30 s sonication using a 40-kHz ultrasonic cleaner in double-distilled water before agroinfiltration [45,48]. Post-infiltration, the leaves underwent cocultivation with *Agrobacterium* with MS medium, as described earlier, for 4 d at 28 °C under light conditions, after which they were utilized for GFP fluorescence content detection. Additionally, following 4 d of cocultivation, part of the infected leaves was transferred to freshly prepared MS medium (supplemented with 300 mg·L^−1^ cefotaxime) for 7, 10, and 15 d, respectively, before being employed for GFP quantitative assessments.

### 2.4. Green Fluorescent Protein (GFP) Microscopy and Quantitative Assays

To meticulously scrutinize and analyze the GFP fluorescence exhibited by mulberry leaves subjected to transformation with the pMV2-*GFP* vector, comprehensive observations were conducted utilizing a Leica DMi8 inverted fluorescence microscope (Leica microsystems, Wetzlar, Germany) by using GFP (450–490 nm excitation, dichroic 495 nm, and 500–550 nm emission) filter sets at 4 d after infiltration. In order to assess the transient expression of *GFP* in mulberry leaves, the quantitative measurement of GFP fluorescent protein content was undertaken using a Plant GFP ELISA Kit (Tianjin Coweino Biotechnology Co., Ltd., Tianjin, China) as per the established procedures described in previous studies [34]. Briefly, mulberry leaf tissues were rapidly frozen in liquid nitrogen and ground separately. 0.1 g of sample was precisely weighed and 800 μL of prechilled PBS (pH = 7.4) was added for extraction for 20 min. The supernatant was separated via centrifugation at 13,000 rpm for 10 min. Standard wells and blank comparison wells were set separately from the testing sample wells, with standard concentrations of 0, 5, 10, 20, 40, 80 ng·mg^−1^. 50 μL of standard solution was added to the standard wells, while 40 μL of sample dilution was added to the testing sample wells. Subsequently, 100 μL of HRP-Conjugate reagent was added to the microELISA strip plate. Blank comparison wells did not contain the sample or the HRP-Conjugate reagent; all other steps were the same. After incubation for 60 min at 37 °C in the dark, the liquid was discarded, and the plate was dried by swinging before adding 20-fold washing buffer to each well. The wells were kept still for 30 s, then drained, and this process was repeated five times before drying by patting. Chromogen solution A (50 μL) and chromogen solution B (50 μL) were added to each well. After reacting in the dark for 15 min at 37 °C, 50 μL of stop solution was added to each well to stop the reaction. The blank well was set to zero, and the absorbance at 450 nm was read using an enzyme-labeler 800 TS microplate reader (BioTek Instruments Inc., Winooski, VT, USA) within 15 min after adding 50 μL of stop solution.

### 2.5. Analysis of Anthocyanin Content

In an endeavor to comprehensively assess the robustness and practical applicability of the meticulously derived transient transformation system, we conducted a series of experiments involving the transient overexpression of *MaANS* [51,52] and *MaDFR* [51,52], genes intricately involved in anthocyanin biosynthesis within mulberry fruits of the ‘Taiguo’ genotype. The coding sequences of *MaANS* and *MaDFR* were amplified from the cDNA of ‘Taiguo’ mulberry fruit by using a pair of primers (Table S2). As previously reported [34], the PCR fragments were then purified and cloned into the pEASYTM-Blunt Cloning Vector (TransGen Biotech, Beijing, China), resulting in plasmids pEASY-*ANS* and pEASY-*DFR*, which were used for verification by sequencing, respectively. And then, the verified plasmids pEASY-*ANS*, pEASY-*DFR* and pMV2-*GFP* were double digested by using *Xba I* and *Kpn I*, respectively. Subsequently, the *Xba I*-*Kpn I* fragment containing *MaANS* and *MaDFR* was cloned into the pMV2 binary vector, resulting in pMV2-*ANS* and pMV2-*DFR*, respectively (Figure S1). The *Agrobacterium* strain LBA4404, harboring distinct constructs, namely pMV2-*GFP*, pMV2-*ANS*, and pMV2-*DFR*, was individually introduced into the mulberry fruits of ‘Taiguo’ harvested at the developmental stage of 10 d after flowering (DAF). As explained earlier, all mulberry fruits, securely placed in glass triangular bottles containing double-distilled water, were subjected to a preliminary sonication lasting 30 s before the subsequent agroinfiltration process. Subsequently, the infiltrated fruits were meticulously incubated in vitro at a controlled temperature of 25 °C for varying durations of 0, 2, 4, 5, and 7 d, as per the previously established protocol [52]. In-depth evaluations of anthocyanin content were executed in strict adherence to a well-established method [52]. Notably, 0.5 g of mulberry fruits was meticulously immersed in 2 mL of a 1% HCL-methanol (1:99, *v*/*v*) solution to facilitate the extraction of anthocyanins. This extraction process was conducted under conditions of controlled darkness for 24 h at a temperature of 4 °C [53]. Using a UV-Vis 2450 spectrophotometer, the absorption spectra at 420–700 nm of the anthocyanin crude extracts were compared to the blank of HCL-methanol (1:99, *v*/*v*) (Shimadzu, Tokyo, Japan). The pigment content was calculated as cyanidin 3-glucoside, using an extinction coefficient of 29,600 L·cm^−1^·mg^−1^ and a molecular weight of 448.8 [53]. Each sample comprised three distinct biological replicates, and for each biological replicate, a minimum of three fruits were sampled, ensuring the reliability and statistical robustness of the experimental data.

### 2.6. Statistical Analysis

The entirety of the data presented in this manuscript underwent meticulous analysis employing one-way ANOVA, followed by a comprehensive examination of differences through Duncan’s multiple range tests [45]. All statistical assays were executed utilizing SPSS 26.0 (IBM, Armonk, NY, USA) with a predefined significance threshold set at *p* < 0.05.

## 3. Results

### 3.1. Transient GFP Expression in the Leaves of Various Mulberry Genotypes

**Figure 1:**
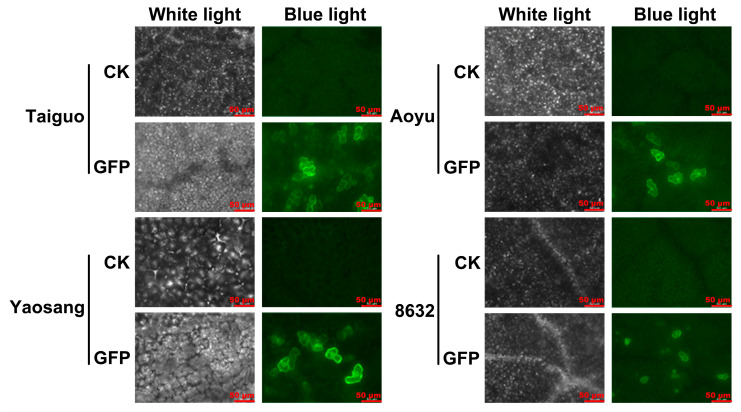
Transient expression status of *GFP* in the leaves of four mulberry genotypes: ‘Taiguo’, ‘Yaosang’, ‘Aoyu’, and ‘8632’. CK indicates non-transformed leaves. GFP denotes leaves transformed with pMV2-*GFP*. The GFP signal was identified at 4 d following agroinfiltration. Scale bar = 50 μm.

In the comprehensive evaluation of the transient expression of the *GFP* reporter gene in mulberry leaves, the pMV2-*GFP* vector was introduced into the leaves of distinct genotypes, namely ‘Taiguo’, ‘Yaosang’, ‘Aoyu’, and ‘8632’, employing *Agrobacterium* strain LBA4404-mediated vacuum infiltration. The findings from the GFP fluorescence examination unveiled a notable absence of any fluorescent signals in non-transformed leaves, except for the inherent auto-fluorescence originating from chloroplasts (Figure 1). In contrast, conspicuous GFP fluorescence signals manifested in the infiltrated leaves across all four mulberry genotypes, scattered at the cellular level, indicating the successful expression of the *GFP* gene in diverse mulberry genotypes (Figure 1). Furthermore, the intensity of the GFP fluorescence signals was notably higher in the ‘Taiguo’ and ‘Yaosang’ leaves compared to other genotypes (Figure 1). The count of cells exhibiting GFP signals was highest in ‘Taiguo’ leaves, with ‘Yaosang’, ‘Aoyu’, and ‘8632’ following in descending order (Figure 1). This detailed assessment provides a nuanced understanding of the differential expression patterns across various mulberry genotypes, offering valuable insights into the transient transformation process.

### 3.2. Improvement of the In Vitro Transient Transformation Systems in Mulberry Leaves

**Figure 2:**
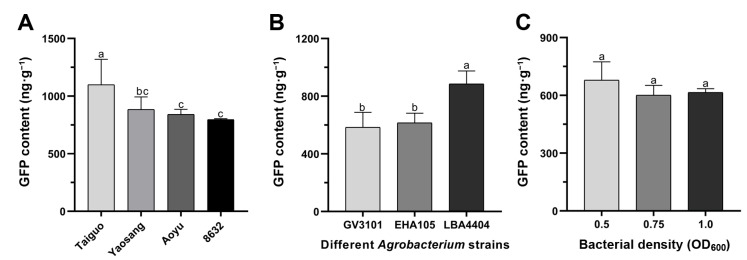
Evaluation of in vitro transient *GFP* expression levels in mulberry leaves at 4 d after infiltration. (**A**) Transient transformation efficiency impacted by diverse mulberry genotypes. (**B**) Transient transformation efficiency influenced by various *Agrobacterium* strains in mulberry leaves of ‘Taiguo’. (**C**) Transient transformation efficiency affected by different bacterial densities in mulberry leaves of ‘Taiguo’. Error bars represent SEs from 10 biological replicates. Means marked with different letters are statistically different based on Duncan’s multiple range tests (*p* < 0.05).

In the pursuit of optimizing the transient transformation system, a thorough examination of various factors influencing the efficiency of transient *GFP* reporter gene expression was conducted. These factors encompassed mulberry genotypes, *Agrobacterium* strain, bacterial density, acetosyringone concentration, sonication duration, and days after infection. Initially, the impact of mulberry genotypes on transient *GFP* gene expression was assessed, revealing that the GFP content in ‘Taiguo’ leaves exceeded that in ‘Yaosang’, ‘Aoyu’, and ‘8632’, demonstrating statistical significance (*p* < 0.05) (Figure 2A). The GFP content was not significantly different among mulberry genotypes ‘Yaosang’, ‘Aoyu’, and ‘8632’ (Figure 2A). Consequently, ‘Taiguo’ mulberry leaves were chosen for further investigating transient transformation in all subsequent experiments. Subsequent comparisons involving agroinfiltration performed with three *Agrobacterium* strains unveiled that mulberry leaves agroinfiltrated with strain LBA4404 exhibited an obviously higher total GFP content than those infiltrated with strains GV3101 and EHA105 in ‘Taiguo’ mulberry leaves (*p* < 0.05) (Figure 2B). There is no significant difference in GFP content between the *Agrobacterium* strains GV3101 and EHA105 (Figure 2B). Consequently, *Agrobacterium* strain LBA4404 was selected for subsequent infiltration tests. And then, the effect of different bacterial densities of strain LBA4404 on transient transformation efficiency was also investigated. The final OD_600_ in the infiltration medium was adjusted to 0.5, 0.75 and 1.0, respectively. The results of GFP content detection indicated that although higher transient transformation efficiency was observed at D_600_ = 0.5, no statisticaly significant differences with those at OD_600_ = 0.75 and OD_600_ = 1.0 were recorded (Figure 2C).

**Figure 3:**
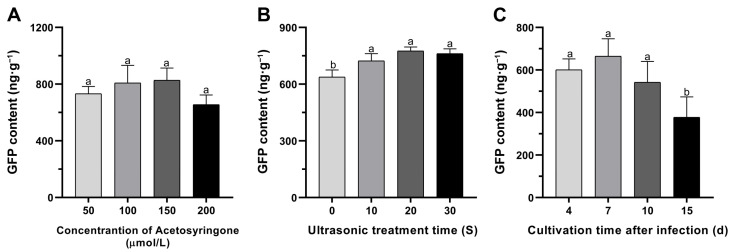
In vitro transient expression levels of *GFP* in mulberry leaves of ‘Taiguo’. (**A**) Transient transformation efficiency driven by different acetosyringone concentrations. GFP fluorescent protein content was undertaken at 4 d after infiltration. (**B**) Transient transformation efficiency caused by different sonication treatments. GFP fluorescent protein content was undertaken at 4 d after infiltration. (**C**) Transient transformation efficiency evaluated by the number of days after agroinfiltration. GFP fluorescent protein content was undertaken at 4, 7, 10 and 15 d after infiltration. Error bars represent SEs from 10 biological replicates. Means with distinct letters indicate significant differences as per Duncan’s multiple range tests (*p* < 0.05).

Furthermore, an in-depth exploration into the dynamic changes of the *GFP* gene’s transient expression levels was conducted, focusing on the variables of acetosyringone concentration, sonication duration, and days after infection (Figure 3). The findings unveiled that the optimal transformation efficiency was achieved with an acetosyringone concentration of 150 μM in the infiltration medium (Figure 3A). Nevertheless, no statistically significant differences in the GFP contents were observed among different acetosyringone concentrations (Figure 3A). Additionally, the outcomes of GFP content detection indicated that various sonication treatments could significantly enhance transient transformation efficiency compared to the control (*p* < 0.05); however, no statistically significant differences were observed between ultrasonic treatments (Figure 3B). Moreover, a comprehensive investigation into transient expression levels of the *GFP* gene was conducted at 4, 7, 10, and 15 d after infection (Figure 3C). Notably, the peak GFP content was observed on the 7th d after infiltration, followed by a gradual decline until the 15th d (Figure 3C). No statistically significant differences were observed at 4, 7, and 10 d after infiltration; only a significant decrease was observed at 15 d after infiltration (Figure 3C).

### 3.3. Transient Overexpression of MaANS and MaDFR in Mulberry Fruits

**Figure 4:**
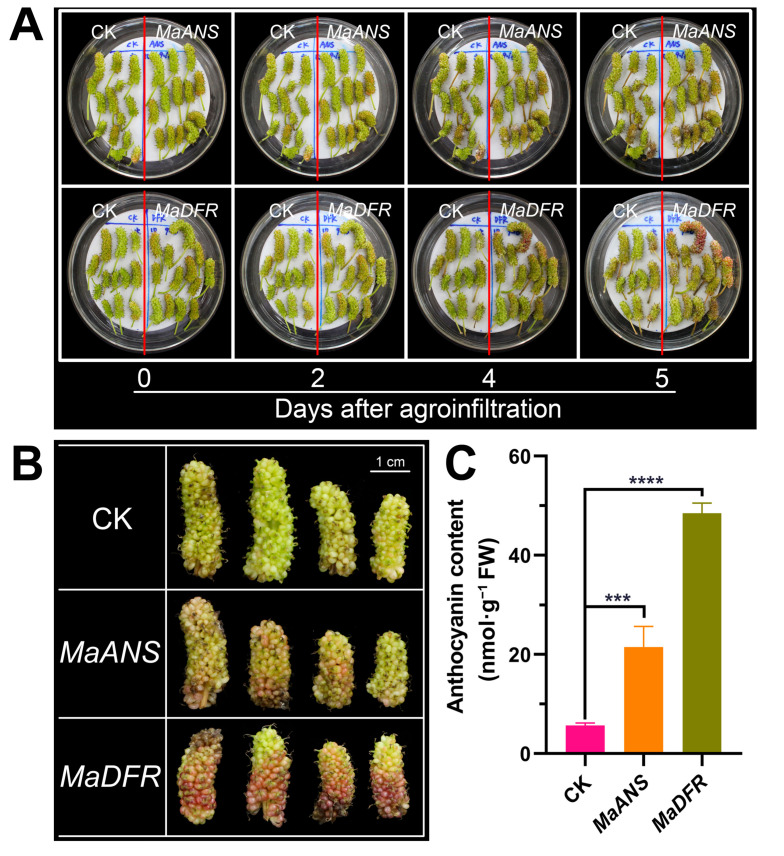
Transient overexpression of *MaANS* and *MaDFR* in ‘Taiguo’ mulberry fruits. (**A**) Morphology of ‘Taiguo’ fruits at various times following agroinfiltration. (**B**) Morphology of ‘Taiguo’ fruits at 7 d after agroinfiltration. (**C**) Examination of the anthocyanin contents in ‘Taiguo’ fruits at 7 d following agroinfiltration. CK, *MaANS*, and *MaDFR* represent fruits transformed with vectors pMV2-*GFP*, pMV2-*ANS*, and pMV2-*DFR*, respectively. Error bars were calculated from the results of three biological replicates, and a minimum of 3 fruits were sampled for each biological replicate. *** and **** represent statistically significant differences based on Duncan’s multiple range tests *p* < 0.01 and *p* < 0.001, respectively.

Moreover, to assess the robustness and dependability of the established transient transformation system utilizing *Agrobacterium*-mediated sonication followed by vacuum infiltration, *MaANS* and *MaDFR* were transiently overexpressed in the mulberry fruits of ‘Taiguo’ at 10 DAF. The results exhibited a substantial increase in fruit coloring at 5 d (Figure 4A) and 7 d (Figure 4B) post-infiltration due to the overexpression of *MaANS* and *MaDFR*, respectively. Furthermore, the anthocyanin content assay disclosed a remarkably significant surge in anthocyanin levels in fruits infiltrated with pMV2-*ANS* and pMV2-*DFR* at 7 d post-agroinfiltration, respectively (Figure 4C). Comparatively, the overexpression of *MaDFR* led to a more pronounced effect on promoting anthocyanin accumulation in the fruits compared to *MaANS* (Figure 4B,C). These findings affirm the involvement of *MaANS* and *MaDFR* in the biosynthesis process and enrichment of anthocyanins in mulberry fruits.

## 4. Discussion

By utilizing *GFP*, a reporter gene, to monitor transformation events, a well-established practice in plant studies [33,34,39,44,45], vacuum infiltration and syringe infiltration have emerged as the most prevalent strategies for *Agrobacterium*-mediated transient transformation [34,45,48,54]. Previous research has reported that a transient transformation assay could be implemented in leaves of mulberry seedlings in vivo by syringe infiltration using *A. tumefaciens* [40]. However, the high density of palisade and spongy mesophyll cells, low density and/or small aperture of stomatal pores, and overall fragility of leaf tissue often result in poor infiltration and mechanical damage from the syringe infiltration [55,56]. When compared with syringe infiltration, vacuum infiltration offers advantages in overcoming challenges posed by the leaf structure and achieving rapid and high-throughput delivery of *Agrobacterium*, resulting in increased infection efficiency and stabilized transformation outcomes. This technique has been successfully applied across various plant species, including persimmon [45], soybean [48], and poplar [33]. In this investigation, the *GFP* gene was introduced into the leaves of four mulberry genotypes (‘Taiguo’, ‘Yaosang’, ‘Aoyu’, and ‘8632’) using *Agrobacterium*-mediated transformation through vacuum infiltration. The results demonstrated clear GFP fluorescence signals in mulberry leaves transformed with pMV2-*GFP* (Figure 1), affirming the successful and transient expression of the *GFP* reporter gene in mulberry leaves, consistent with prior reports [40]. Variations in leaf tissue structure among different mulberry genotypes might contribute to inconsistent transformation efficiency, as reported in previous studies, indicating varying receptiveness to *Agrobacterium* among different plant genotypes [48,57,58]. The intensity of GFP fluorescence signals and the number of cells exhibiting GFP signals were notably higher in ‘Taiguo’ leaves, followed by ‘Yaosang’, ‘Aoyu’, and ‘8632’ (Figure 1). Correspondingly, the GFP content determination results followed a similar trend of level change (Figure 2A).

Factors such as high density of palisade and spongy mesophyll cells, small aperture of stomatal pores in leaf tissue, and/or low density usually lead to poor *Agrobacterium* delivery and reduced transformation efficiency [59,60]. Sonication, as reported in previous studies, induces microwounds in the plant tissue, enhancing *Agrobacterium* delivery and allowing more efficient bacterial access to internal tissues [46,47]. The sonication treatments could significantly enhance transient transformation efficiency than the untreated control (*p* < 0.05), but no statistically significant differences were recorded between sonication treatments (Figure 3B). Similar results have also been observed in soybean, in which vacuum infiltration after 30 s of sonication led to significantly greater expression of GUS than vacuum application alone [48]. Though sonication treatment increased the efficiency of agroinfiltration, the mechanical damage resulting from sonication also caused necrosis in leaf tissue [48]. This may also explain why the transient transformation efficiency of 30 s sonication treatment is lower than that of 20 s sonication treatment, as shown in the data (Figure 3B). Although sonication and vacuum infiltration have been used together for *Agrobacterium*-mediated transformation of plant tissues and plants [61,62], these treatments have not been previously evaluated for transient transformation in mulberry seedlings in vitro.

Additionally, the impact of different *Agrobacterium* strains and bacterial densities on the transformation efficiency was assessed in vitro in mulberry leaves of ‘Taiguo’ (Figure 2B,C). Although *Agrobacterium* strains GV3101, EHA105, and LBA4404 are commonly used [40,48], differential transformation rates were observed between different *Agrobacterium* strains in this study, as previously reported. The *Agrobacterium* strain LBA4404 exhibited significantly higher total GFP content in mulberry leaves (*p* < 0.05), compared to strains EHA105 and GV3101 (Figure 2B). Notably, higher transformation efficiency was observed at OD_600_ 0.5, compared to OD_600_ 0.75 and OD_600_ 1.0 (Figure 2C), highlighting that high-density *Agrobacterium* can potentially disrupt the physiological functions of plant cells or even lead to cell death. Similar findings have been reported, emphasizing that the infectivity of *A. tumefaciens* strain LBA4404 was stronger than that of GV2260 and A281 in *M. indica* cv. K2 [30], and the level of OD_600_ 0.5 was chosen for consistent transient expression of the *GUS* gene in mulberry seedlings (*M. alba* L.) [40]. Many different strains of *Agrobacterium* are available and may show additional potential for agroinfiltration or transformation of various plant species [63]. These findings underscore the necessity of optimizing mulberry genotypes, *A. tumefaciens* strain, and bacterial density for an efficient transient transformation system.

Acetosyringone, a phenolic signal known to enhance *Agrobacterium*’s ability to transform host plants and improve transformation efficiency, is a key factor for successful transformation [59]. Four concentrations of acetosyringone (50, 100, 150 and 200 μM) in the infiltration medium were investigated in this study. The results of GFP content detection indicated that although a higher transient transformation efficiency was observed at 150 μM acetosyringone, no statisticaly significant differences with other concentrations were recorded (Figure 3A). The results exhibited an increase in transformation efficiency with increasing acetosyringone concentration, reaching its highest at 150 μM, and then decreasing at 200 μM (Figure 3A). Comparable outcomes and trends have been reported in previous studies involving persimmon [34] and banana [60]. Typically, the expression of foreign genes can be detected within 12 h after infection and lasts for 3–4 d [34]. The results revealed that the foreign genes are either lost via cell division or included into the host plant genome. The findings demonstrated that *GFP* expression levels can be observed throughout the 4–15 d after infiltration and the levels of GFP content remained unchanged from 4 to 10 d after infiltration, and then remarkably decreased at 15 d after infiltration (Figure 3C). Comparable results have been identified in persimmon [34], grapes [36], and tobacco [64]. 

To assess and characterize the stability and reliability of the transient transformation system employing sonication followed by vacuum infiltration, the *MaANS* [51,52] and *MaDFR* [51,52] involved in anthocyanin accumulation were inserted into mulberry fruits of the ‘Taiguo’ for transient overexpression function verification in vitro. Compared to the control fruit transformed with pMV2-*GFP*, the overexpression of *MaANS* and *MaDFR* contributed to significantly enhancing fruit coloration (Figure 4A,B) and led to a substantially increased level of anthocyanins in mulberry fruits (Figure 4C), respectively. The overexpression of *MaDFR* could better promote the accrual of anthocyanins compared to *MaANS* (Figure 4). These findings further confirmed that *MaANS* and *MaDFR* are critical individual genes involved in the biosynthesis and accumulation of anthocyanins within mulberry fruits, as reported in prior studies [51,52,65]. Taken together, our modified transient transformation approach can be suitable for transient overexpression of heterologous genes and functional assessment and analysis in mulberry.

## 5. Conclusions

Utilizing *GFP* as a reliable reporter gene, we have successfully devised an uncomplicated, highly efficient, and stable transient transformation system in mulberry leaves in vitro, by employing *A. tumefaciens*-mediated transformation with subsequent sonication and vacuum infiltration. More importantly, this system was successfully used for the transient overexpression of *MaANS* and *MaDFR* in mulberry fruits in vitro, leading to a substantial increase in both fruit coloring and anthocyanin levels. In all, this optimized transient transformation system not only showcases its versatility in gene functional analysis but also establishes a foundation for further advancements in post-genomic studies of mulberry trees.

## Data Availability

All data are available in the manuscript or the Supplementary Materials.

## Supplementary Materials

The following supporting information can be downloaded at: https://www.mdpi.com/article/10.3390/genes15101277/s1, Figure S1: The T-DNA region of the binary plant vector. The T-DNA region of pMV2-GFP showing left (LB) and right (RB) border sequences, 35S promotor (35S), *GFP* coding sequence (GFP), *MaANS* coding sequence (MaANS), *MaDFR* coding sequence (MaDFR), octopine synthase polyadenylation signal (OCS), and the coding region for the neomycin phosphotransferase gene (NosP-NPTII); Table S1: Optimization of experimental parameters; Table S2: Sequences of the primers.
