# Supplementary material for: Refinement and Enhancement of Agrobacterium-Mediated Transient Transformation for Functional Gene Examination in Mulberry (Morus L.)
Source: Genes (Basel). 2024 Sep 28;15(10):1277. doi: 10.3390/genes15101277 (PMC11507023; doi:10.3390/genes15101277)
Supplement: Supplementary file 1 [file genes-15-01277-s001.zip › genes-3216693-supplementary.pdf]

Supplementary Table S1 Optimization of experimental parameters

**Factors**	**Parameters**	**Infiltration medium and conditions**	**Experimental subject**	**Cocultivation condition**	**Sampling time**
Agrobacterium tumefaciens strains	GV3101<br>EHA105<br>LBA4404	10 mM MES, and 10 mM $MgCl_2$, 150 μM acetosyringon, OD_{600} 0.75 of *Agrobacterium tumefaciens* strains (GV3101, EHA105 and LBA4404), supplemented with the surfactant 0.02% Silwet L-77, with 30 s sonication followed by 20 min vacuum infiltration (0.07 MPa).	Leaves of mulberry cultivar 'Taiguo'	MS medium (pH 5.8, composed of 6.5 g·L^{-1} agar, 1.0 mg·L^{-1} 6-BA, 30 g·L^{-1} sucrose, and 0.5 mg·L^{-1} NAA), for 4 d at 28 °C	4 d after agroinfiltration
Bacterial density of *Agrobacterium tumefaciens* LBA4404,	OD_{600} 0.5, 0.75 and 1.0	10 mM MES, and 10 mM $MgCl_2$, 150 μM acetosyringon, OD_{600} (0.5, 0.75 and 1.0) of *Agrobacterium tumefaciens* LBA4404, supplemented with the surfactant 0.02% Silwet L-77, with 30 s sonication followed by 20 min vacuum infiltration (0.07 MPa).	Leaves of mulberry cultivar 'Taiguo'	MS medium (pH 5.8, composed of 6.5 g·L^{-1} agar, 1.0 mg·L^{-1} 6-BA, 30 g·L^{-1} sucrose, and 0.5 mg·L^{-1} NAA), for 4 d at 28 °C	4 d after agroinfiltration
Mulberry genotype	'Taiguo'<br>'Yaosang'<br>'8632'<br>'Aoyu'	10 mM MES, and 10 mM $MgCl_2$, 150 μM acetosyringon, OD_{600} 0.5 of *Agrobacterium tumefaciens* LBA4404, supplemented with the surfactant 0.02% Silwet L-77, with 30 s sonication followed by 20 min vacuum infiltration (0.07 MPa).	Leaves of four mulberry genotypes: 'Taiguo' 'Yaosang' '8632' and 'Aoyu', respectively	'Taiguo', '8632' and 'Aoyu' were maintained on MS medium (pH 5.8, composed of 6.5 g·L^{-1}L agar, 1.0 mg·L^{-1} 6-BA, 30 g·L^{-1} sucrose, and 0.5 mg·L^{-1} NAA), for 4 d at 28 °C. 'Yaosang' was maintained on DKW medium (pH 5.8, composed of 30 g·L^{-1} sucrose, 3.0 mg·L^{-1} ZT, 3.0 mg·L^{-1} 6-BA, 0.5 mg·L^{-1} NAA and 6.5 g·L^{-1} agar), for 4 d at 28 °C.	4 d after agroinfiltration

Acetosyringone concentration	50, 100, 150 and 200 μM	10 mM MES, and 10 mM $MgCl_2$, acetosyringon (50, 100, 150 and 200 μM), OD_{600} 0.5 of *Agrobacterium tumefaciens* LBA4404, supplemented with the surfactant 0.02% Silwet L-77, with 30 s sonication followed by 20 min vacuum infiltration (0.07 MPa).	Leaves of mulberry cultivar 'Taiguo'	MS medium (pH 5.8, composed of 6.5 g·L^{-1} agar, 1.0 mg·L^{-1} 6-BA, 30 g·L^{-1} sucrose, and 0.5 mg·L^{-1} NAA), for 4 d at 28 °C	4 d after agroinfiltration
Sonication time	0, 10, 20 and 30 s	10 mM MES, and 10 mM $MgCl_2$, 150 μM acetosyringon, OD_{600} 0.5 of *Agrobacterium tumefaciens* LBA4404, supplemented with the surfactant 0.02% Silwet L-77, with sonication (0, 10, 20 and 30 s) followed by 20 min vacuum infiltration (0.07 MPa).	Leaves of mulberry cultivar 'Taiguo'	MS medium (pH 5.8, composed of 6.5 g·L^{-1} agar, 1.0 mg·L^{-1} 6-BA, 30 g·L^{-1} sucrose, and 0.5 mg·L^{-1} NAA), for 4 d at 28 °C	4 d after agroinfiltration
Days after infection	4, 7, 10 and 15 d	10 mM MES, and 10 mM $MgCl_2$, acetosyringon (50, 100, 150 and 200 μM), OD_{600} 0.5 of *Agrobacterium tumefaciens* LBA4404, supplemented with the surfactant 0.02% Silwet L-77, with 30 s sonication followed by 20 min vacuum infiltration (0.07 MPa).	Leaves of mulberry cultivar 'Taiguo'	MS medium (pH 5.8, composed of 6.5 g·L^{-1} agar, 1.0 mg·L^{-1} 6-BA, 30 g·L^{-1} sucrose, and 0.5 mg·L^{-1} NAA), for 4 d at 28 °C. Following 4 d of cocultivation, part of the infected leaves were transferred to freshly prepared MS medium (supplemented with 300 mg·L^{-1} cefotaxime) for 7, 10, and 15 d, respectively.	4, 7, 10 and 15 d after agroinfiltration

Supplementary Table S2 Sequences of the primers

**Primer Name**	**Sense primer (5' to 3')**	**Antisense primer (5' to 3')**
DFR	TAGCTAACGGTACGTTTGGTAGTGA	ATTGCTGCTAATCTTTTGAAAGACA

ANS	TTGAAACAAGGAGGGTTAAACTCA	ATTAGCCTAATTTGTACCACTACTTTTC

[a]From Li et al. (2020)[47]

Supplementary Figure S1 The T-DNA region of the binary plant vector. The T-DNA region of pMV2-*GFP* showing left (LB) and right (RB) border sequences, 35S promotor (35S), *GFP* coding sequence (GFP), *MaANS* coding sequence (MaANS), *MaDFR* coding sequence (MaDFR), octopine synthase polyadenylation signal (OCS), and the coding region for the neomycin phosphotransferase gene (NosP-NPTII).

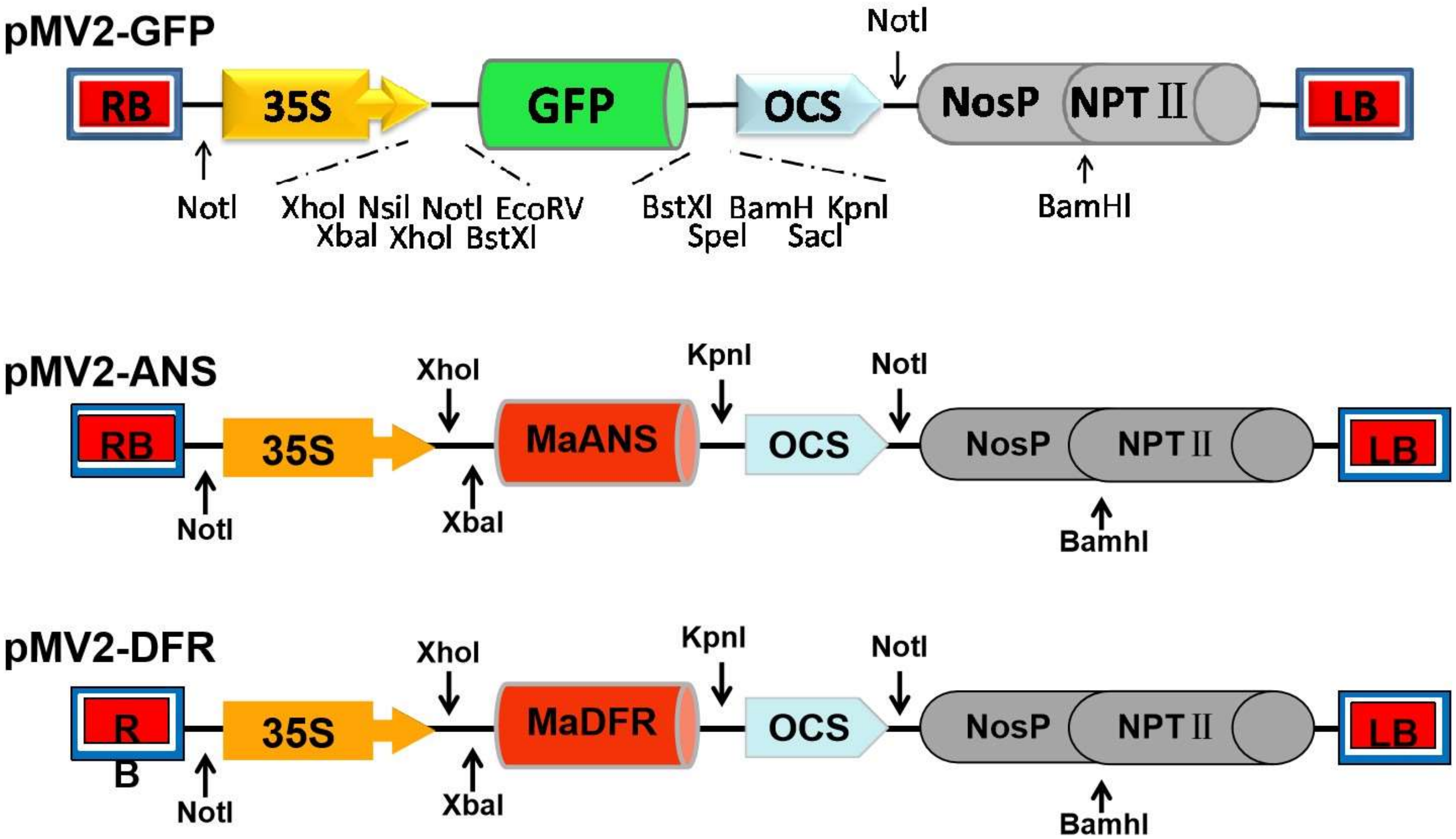
pMV2-GFP
RB
35S
GFP
OCS
NotI
NosP
NPT II
LB
NotI
XhoI NsiI NotI EcoRV
XbaI XhoI BstXI
BstXI BamH KpnI
SpeI SacI
BamHI
pMV2-ANS
XhoI
KpnI
NotI
RB
35S
MaANS
OCS
NosP
NPT II
LB
NotI
XbaI
Bamhl
pMV2-DFR
XhoI
KpnI
NotI
R
B
35S
MaDFR
OCS
NosP
NPT II
LB
NotI
XbaI
Bamhl
